# Effects of a genetically modified potato on a non-target aphid are outweighed by cultivar differences

**DOI:** 10.1007/s10340-017-0831-6

**Published:** 2017-01-18

**Authors:** Jenny Lazebnik, Salvatore Arpaia, Ferdinando Baldacchino, Paolo Banzato, Stefania Moliterni, Jack H. Vossen, Els M. van de Zande, Joop J. A. van Loon

**Affiliations:** 1 Wageningen University and Research, Entomology, Wageningen, The Netherlands; 2 ENEA, Trisaia Research Centre, Rotondella, Matera Italy; 3 Wageningen University and Research, Plant Breeding, Wageningen, The Netherlands

**Keywords:** Genetic modification, Non-target testing, Greenhouse, Environmental risk assessment, *Phytophthora infestans*, *Solanum tuberosum*, *Myzus persicae*

## Abstract

Insect–plant interactions may be unintentionally affected when introducing genetically modified (GM) crops into an agro-ecosystem. Our aim was to test the non-target effects of a late blight-resistant GM potato on *Myzus persicae* in greenhouse and climate room experiments and understand how position and number of R gene insertions can affect non-targets in GM events. We also aimed to compare results to baseline differences among three conventional potato varieties varying in resistance to late blight. Aphid development and survival were affected by some GM events in the first generation, though effects disappeared in the second generation. Effects were not dependent on the presence of a marker gene or the insertion of a second resistance gene. Positional effects of gene insertion influenced aphid performance on certain GM events. However, aphid fitness varied considerably more between conventional potato varieties than between Désirée and the GM events. Comparing different GM events to the non-transformed variety is relevant, since unintended effects of insertion can occur. Our protocols can be recommended for *in planta* risk assessments with aphids. Ecological perspective is gained by selecting several measured endpoints and by comparing the results with a baseline of conventional cultivars.

## Key message

- We investigated the hypothesis that characteristics of a GM potato may influence the non-target aphid *Myzus persicae*.
- Aphid performance was affected by the resistance gene position but not by the number of resistance genes or the presence of an antibiotic resistance marker gene.
- Aphid performance varied considerably more between conventional cultivars than between the unmodified and the GM potato.
- These findings support future protocols for risk assessments of GM crops on non-target insects.

## Introduction

To be considered for cultivation in agriculture, genetically modified (GM) crops must be subject to environmental risk assessment (ERA). The biodiversity and ecology of organisms in the agro-ecosystem are considered important in ERA. Plants are the primary producers supporting the trophic webs of agro-ecosystems, and the direct and indirect consequences of introducing genetically modified crops are therefore a relevant concern (Arpaia 2010; EFSA 2010). Risk assessments should be done in several stages or tiers, starting with experiments that have a high likelihood of detecting effects on non-targets to more complex and realistic field conditions (Andow and Hilbeck 2004; Andow and Zwahlen 2006; Houshyani 2012; Kos et al. 2009; Romeis et al. 2011). Each consecutive tier in the ERA should use the feedback acquired in previous steps. Trials in confined conditions are important in early tiers of ERA to establish whether direct effects occur on the life history of particularly important members of the agro-ecosystem or representatives of important functional groups (Andow et al. 2013; Birch et al. 2007; Houshyani 2012; Romeis et al. 2011, 2013).

Before the introduction of GM plants into the ecosystem, testing for non-target effects of a GM crop in the greenhouse first requires a thorough and transparent selection of appropriate non-target organisms (NTOs) (Carstens et al. 2014; EFSA 2010). These tests should be reproducible and reliable and are an important step in the ERA process. A selection procedure of relevant functional groups and endpoints to test must also be included in the ERA. In this study, we based the selection on the protocol outlined in the EFSA guidance document on ERA of GM plants (EFSA 2010) as well as on several other sources (Andow et al. 2013; Gillund et al. 2013; Romeis et al. 2013, 2014; Scholte and Dicke 2005). We selected the aphid *Myzus persicae* Sulzer to test *in planta* the non-target effects of a genetically modified potato expressing resistance to late blight.

Most conventional potato cultivars are susceptible to late blight which is caused by the widespread pathogen *Phytophthora infestans* (Mont.) de Bary, a hemibiotrophic oomycete which colonizes potato leaves, stems and tubers. Genetic modification of the cultivar Désirée conferred resistance to *P. infestans* through the insertion of one or two resistance genes (*R* genes) from crossable potato (*Solanum tuberosum* L.) relatives, *Solanum venturii* Hawkes & Hjert., *(Rpi*-*vnt1*), and *Solanum stoloniferum* Schltdl & Bouché (*Rpi*-*sto1*) (Haesaert et al. 2015; Haverkort et al. 2016). *R* genes code for receptor proteins which recognize distinct pathogen effectors (in this case from *P. infestans*). This recognition initiates signal transduction cascades leading to callose deposits and cell death in infected and surrounding cells preventing the pathogen from further spread, which is macroscopically visible as a hypersensitivity response (HR) (Kamoun et al. 1999; Vleeshouwers et al. 2000, 2011).

Late blight *R* genes can be co-inserted with a selectable marker gene from a bacterium coding for resistance to an antibiotic (transgenesis) or using a marker-free transformation protocol. Because the *R* genes used in this study are derived from crossable species and the transformation events contain no ‘foreign’ DNA, the latter protocol is referred to as cisgenesis. We tested two transgenic and two cisgenic events containing the same single *R* gene (*Rpi*-*vnt1*). Also we tested two transgenic events harbouring two *R* genes (*Rpi*-*vnt1* and *Rpi*-*sto1*). The location of the *R* gene insertion in the genome may have an impact on other plant functions and indirectly on non-target aphids. By testing two transformation events of each construct, position effects could be assessed. We also assessed the reproducibility of the experimental protocol by performing the assays on the same plant clones in two laboratories each maintaining their own *M. persicae* colonies.

In order to compare the magnitude of the effects of these modifications with the variation among commercially available conventional potato varieties, we compared a cisgenic event (also used in concurrent field experiments) with four conventional varieties (including Désirée) varying in their susceptibility to *P. infestans* (Table 1).

**Table 1:** Characteristics of genetically modified events and cultivars used in this study

Event/cultivar	Event type	Resistance rating to *Phytophthora* on foliage	*R* gene insertion, wild relative	Marker gene
A15-31	Cisgenic	Very high	*Rpi*-*vnt1*, *Solanum venturii*	None
A15-84	Cisgenic	Very high	*Rpi*-*vnt1*, *Solanum venturii*	None
A15-45^b^	Cisgenic	Very high	*Rpi*-*vnt1*, *Solanum venturii*	None
A13-13	Transgenic	Very high	*Rpi*-*vnt1*, *Solanum venturii*	NPTII (kanamycin resistance)
A13-17	Transgenic	Very high	*Rpi*-*vnt1*, *Solanum venturii*	NPTII (kanamycin resistance)
A16-02	Stacked transgenic	Very high	*Rpi*-*vnt1*, *Solanum venturii,* and *Rpi*-*sto1*, *Solanum stoloniferum*	NPTII (kanamycin resistance)
A16-24	Stacked transgenic	Very high	*Rpi*-*vnt1*, *Solanum venturii,* and *Rpi*-*sto1*, *Solanum stoloniferum*	NPTII (kanamycin resistance)
Désirée	Isogenic, conventional	Low–medium^a^	None	None
Bintje	Conventional	Low^a^	None	None
Première	Conventional	Low–medium^a^	None	None
Sarpo Mira	Conventional	Very high^a^	None	None

^a^Rating taken from the European Cultivated Potato Database (ECPD 2015)

^b^Not used for Figs. 1 and 3 due to restricted availability at the time of experiment

### Selection of non-target species *Myzus persicae* for *in planta* testing

Many species may be exposed to GM plants in any agro-ecosystem. Since not all species can be tested, a representative subset of NTOs should be selected for consideration in the risk assessment of each GM plant. The GMO Panel of the European Food Safety Authority (EFSA) proposes a species selection approach (EFSA 2010). *M. persicae* Sulzer (Hemiptera: Aphididae) was chosen based on a final ranking using the aforementioned approach, which includes several important factors. First, it is listed as the most collected phloem feeder in the EFSA arthropod database (Riedel et al. 2016) and second most collected species on potato giving it high relevance as a focal NTO. Second, the species is amenable for rearing in many laboratories, which allows for the measurement of survival and intrinsic rate of increase, which can be used to estimate the population dynamics of this pest.

Aphids are the most important insect pests of potato (Meissle et al. 2012; Radcliffe 1982), and the polyphagous *M. persicae* is the most prevalent and studied among those. Aphids cause direct damage through piercing and sucking from the plant’s phloem. More problematic is the fact that *M. persicae* is a vector of over one hundred plant viruses, with about twelve directly affecting potato crops, including several leaf-roll viruses (Kennedy et al. 1962; Ng and Perry 2004; Van Emden et al. 1969). Aphids are a major prey species host many parasitoids (Müller et al. 1999) and are prey to predators such as larval syrphid flies (Raj 1989), ladybugs (Majerus 1994), lacewings, spiders and others (Van Emden et al. 1969). Despite the specificity of an *R* gene for resistance against *P. infestans*, it is nevertheless important to understand whether the modification can affect the behaviour or performance of an important NTO like *M. persicae* (Han et al. 2016) and its population dynamics.

## Experimental procedures

### Plant material

The GM events tested in this study were developed by the Laboratory of Plant Breeding of Wageningen University and Research (Haesaert et al. 2015; Haverkort et al. 2016). They have been created using *Agrobacterium tumefaciens*-mediated transfer of the native *Rpi*-*vnt1* gene, from *Solanum venturii*, using marker-assisted (events A13-13, 17) and marker-free transformation methods (events A15-31, 45, 84). Also, two marker-assisted transformation events (A16-02 and A16-24) were used that were generated using a single T-DNA harbouring the native *Rpi*-*vnt1* and *Rpi*-*sto1* (from *Solanum stoloniferum*) genes. The tested conventional cultivars and GM events (defined here as clones with gene insertions conferring resistance to the target *P. infestans*) are described in Table 1. Events were selected as apparently ‘true to type’ as they were morphologically indistinguishable from non-transformed Désirée under tuber-sown field conditions (Haverkort et al. 2016).

All GM events and conventional cultivars were maintained in vitro, on agar medium (purified agar 0.8% + 2.2 g/L Murashige & Skoog + Duchefa 4.4 g/L + saccharose 20 g/L + micro-agar 8 g/L; pH 5.8) in sterile containers. Containers were kept in a climate room at 16:8 light/dark conditions, 21 °C during light hours and 15 °C when dark, and 70% relative humidity. Cuttings were transplanted five weeks before the experiments to allow for root growth, seedlings then transplanted to larger pots and allowed to grow for five weeks before being used in experiments.

### Aphid rearing and experimental set-up

#### WUR

*Myzus persicae* were collected in 2004 from Wageningen, The Netherlands (51°59′11.5″N 5°39′48.4″E), and reared at the Laboratory of Entomology, Wageningen University and Research (WUR). They were originally kept on radish but maintained for several generations on *S. tuberosum* cultivar Désirée before experiments began under the same climate room conditions described above.

#### ENEA

The colony was started from a laboratory strain originally reared at the University of Bologna. The strain was maintained on *S. tuberosum* cultivar Désirée for several generations before experiments began. The *M. persicae* colony was maintained under 16:8 light/dark conditions, 24 °C during light hours and 18 °C when dark, and 70% relative humidity.

### Testing the GM potato events and conventional potato varieties

First we tested the intrinsic rate of increase and survival of aphids between the non-transformed Désirée and the following GM (from Désirée) events: A15-31, A15-45 (both cisgenic), A13-13, A13-17 (both transgenic), A16-02 and A16-24 (both transgenic with two R genes); all events are described in Table 1. Then, to test reproducibility, WUR and ENEA performed similar experiments comparing specifically the cisgenic events A15-31 and A15-45 to the non-transformed Désirée. Lastly, we compared several conventional potato cultivars: Désirée, Bintje, Première and Sarpo Mira (described in Table 1) with the same measured endpoints as for the aforementioned experiments.

One-day-old aphid nymphs were used in each experiment. Aphid nymphs were placed singly in clip cages (25 mm diameter; 10 mm high) on the abaxial surface of two (at ENEA) or three leaves (WUR) on each plant. Ten (at WUR) to fifteen (at ENEA) plant replicates of each event and the non-transformed Désirée cultivar were used and randomly distributed in the climate room. Due to space limitations, this was split into two or three rounds, each round testing five plants from each event and non-transformed Désirée.

We monitored the fitness of *M. persicae* for two generations. Aphids were checked every day for mortality and for offspring production; neonate nymphs were counted and removed daily. At WUR, once the first generation produced its first nymphs, one of these was caged on another leaf of the same plant; at ENEA second generations were transferred to a new plant. The parameters collected were: pre-reproductive period and total fecundity, for calculation of intrinsic rate of increase (*R*_m_) and aphid mortality of both generations. Intrinsic rate of increase was calculated as described in Wyatt and White (1977): *R*_m_ = 0.74 (ln Md)/*d*, where Md is the effective fecundity and *d* the length of the pre-reproductive period. The means for all aphid parameters used to calculate survival and intrinsic rate of increase are documented in Appendix of Tables 2, 3 and 4.

**Table 2:** Aphid fitness parameters used to quantify aphid intrinsic population increase [mean and standard error (SE)] for the experiments at WUR and ENEA on genotypes Désirée, A15-31, A15-45

Laboratory	Genotype	Generation	Intrinsic rate of increase (*R*_m_)	SE	Effective fecundity (Md)	SE	Pre-reproductive period in days (d)	SE	Survival time (d)	SE
ENEA	Désirée	1	0.22	0.03	11.04	1.89	7.00	0.43	20.64	1.63
		2	0.30	0.04	11.38	2.85	5.38	0.53	18.70	3.30
	A15-31	1	0.29	0.02	11.79	1.59	6.00	0.49	19.86	1.53
		2	0.37	0.01	21.00	2.55	6.00	0.38	16.27	3.46
	A15-45	1	0.22	0.02	12.38	1.58	7.57	0.33	18.00	1.54
		2	0.33	0.06	9.75	1.47	6.00	0.91	20.45	3.22
Wageningen	Désirée	1	0.26	0.02	17.76	2.38	7.86	0.33	21.29	2.40
		2	0.22	0.01	14.75	1.33	9.72	0.46	21.95	2.00
	A15-31	1	0.32	0.01	25.56	1.63	7.60	0.21	23.32	2.50
		2	0.25	0.01	20.17	1.61	8.84	0.28	23.60	1.75
	A15-45	1	0.26	0.01	22.58	1.94	8.70	0.36	24.04	1.97
		2	0.24	0.01	18.89	2.35	9.32	0.56	25.05	1.78

**Table 3:** Aphid fitness parameters measured for *R*_m_ calculations at WUR for experiments on all other GM events

Genotype	Generation	Intrinsic rate of increase (*R*_m_)	SE	Effective fecundity (Md)	SE	Pre-reproductive period in days (d)	SE	Survival time (d)	SE
Désirée	1	0.29	0.01	24.00	1.52	8.14	0.23	20.14	1.14
	2	0.34	0.03	30.60	3.85	7.60	0.81	17.60	1.86
A15-31	1	0.32	0.01	29.27	2.40	7.87	0.19	20.27	1.04
	2	0.34	0.01	35.00	4.50	7.70	0.33	19.40	0.90
A15-84	1	0.30	0.01	27.83	2.44	8.00	0.18	20.67	0.82
	2	0.31	0.02	28.45	3.84	8.09	0.31	19.64	0.68
A16-02	1	0.31	0.01	27.19	1.97	7.95	0.15	21.29	1.02
	2	0.32	0.01	26.44	2.44	7.78	0.22	19.11	1.23
A16-24	1	0.31	0.01	29.75	2.24	8.06	0.25	20.94	1.39
	2	0.31	0.02	28.86	4.56	8.00	0.44	19.00	1.59
A13-13	1	0.31	0.01	30.37	2.30	8.11	0.15	22.21	1.27
	2	0.34	0.01	34.47	3.40	7.80	0.31	18.47	0.90
A13-17	1	0.32	0.01	29.11	2.17	7.72	0.16	19.67	0.97
	2	0.34	0.01	33.22	4.90	7.44	0.41	19.78	1.79

**Table 4:** Aphid fitness parameters measured for *R*_m_ calculations for parameters measured in baseline comparisons

Genotype	Intrinsic rate of increase (*R*_m_)	SE	Effective fecundity (Md)	SE	Pre-reproductive period in days (d)	SE	Survival time (d)	SE
Désirée	0.27	0.01	30.50	1.77	9.25	0.21	19.90	1.62
A15-31	0.25	0.02	28.43	3.53	9.50	0.20	16.25	1.86
Bintje	0.22	0.01	17.38	1.71	9.44	0.16	17.06	1.54
Premiere	0.16	0.01	11.00	1.95	10.82	0.30	13.33	1.45
Sarpo Mira	0.08	0.02	4.38	1.24	10.75	0.53	10.32	1.22

The same methodology was applied to a second experiment in a greenhouse comparing the first generation of aphid life-history parameters on one cisgenic event (A15-31, highly resistant) and four conventional cultivars varying in their foliar resistance to *P. infestans*. Cultivar Bintje has a resistance rating of low to very low, cultivar Première and Désirée rate low to medium and Sarpo Mira rates highly resistant to *P. infestans* (ECPD 2015).

### Statistical analysis

Based on a preliminary small-scale experiment (15 individuals), we conducted a prospective power analysis. The measurement endpoint selected was the length of the pre-reproductive period for which a standard deviation of 1.7 days was found. We added a safety margin and set the standard deviation in the power analysis to be 1.9 days. The common within-group standard deviation was set at 2.5, based on the variability registered in the actual experiment. This effect was selected as the smallest relevant effect. The criterion for significance (alpha) was set at 0.050 for a two-tailed test. The analysis was conducted using the Power and Precision 2.1 software (Borenstein et al. 2001). The results indicated a sample size of 28 individuals for each group, and the study will have power of 81.3% to yield a statistically significant result for the differences indicated.

Intrinsic rate of increase was tested with a mixed linear model or generalized linear mixed model when data did not meet the assumptions of normality, with fixed factors being ‘potato event’ and ‘aphid generation’ and random factors including the ‘plant or pot number’ (since there were three clip cages per plant), nested within ‘round’ (experiment was replicated in two rounds). The model was chosen by backwards selection comparing AIC values of simpler models (Burnham et al. 2010). The fixed factor ‘aphid generation’ (first or second generation) proved to have an influence on aphid intrinsic rates of increase (*p* = 0.0034). For some events, there was an interaction effect between ‘generation’ and the ‘potato event’. For this reason, we separated the two aphid generations and used separate models for each using the same random factors as above. Analysis for comparisons to baseline cultivars was done in a similar way as above, though the experiment was conducted in one round, for one aphid generation, and the only random effect included in the model was ‘plant or pot number’. Analyses for intrinsic rates of increase were conducted using R Statistical Software (R Core Team 2014), with the ‘nlme’ package.

Survival analyses were conducted using a Cox proportional hazards regression model. This was also separated by generation, which played an important role in aphid survival (*p* = 0.0005) and interacted with the fixed effect of ‘potato event’. This model included the same nested random effects as above and was performed using R Statistical Software (R Development Core Team 2014), with the ‘survival’ package.

## Results

### Désirée compared to GM events

#### Comparison of events

**Fig. 1:**
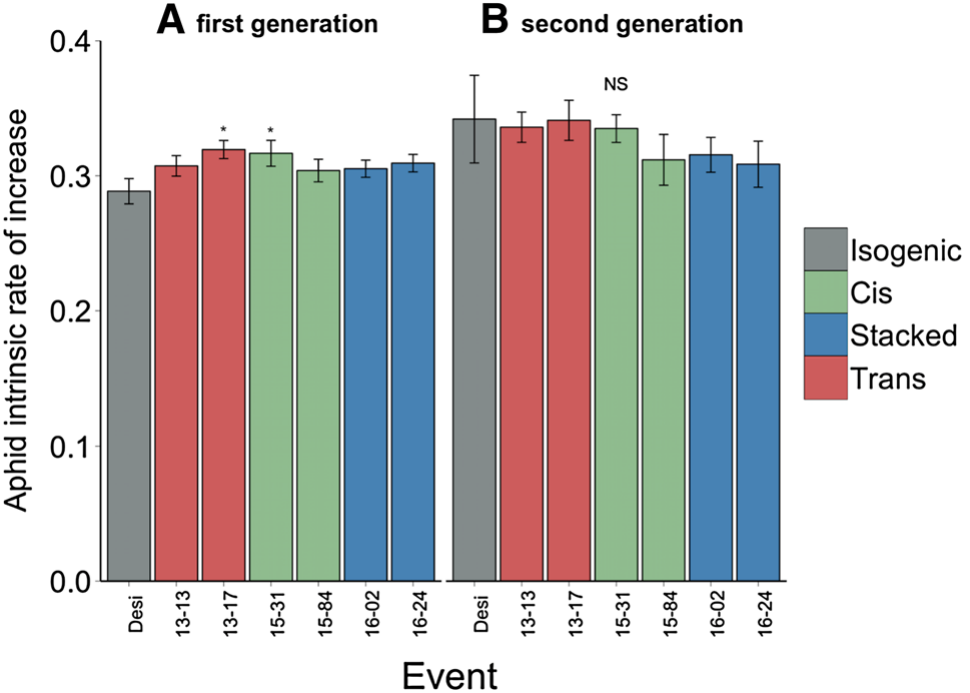
Mean aphid intrinsic rate of increase (±SE) on *Solanum tuberosum* isogenic cultivar Désirée, compared to several genetically modified events for two aphid generations. Two events of cisgenic, transgenic and stacked transgenic potatoes were compared. *Asterisk* (*) indicates significant differences from the isogenic cultivar within the generation

**Fig. 2:**
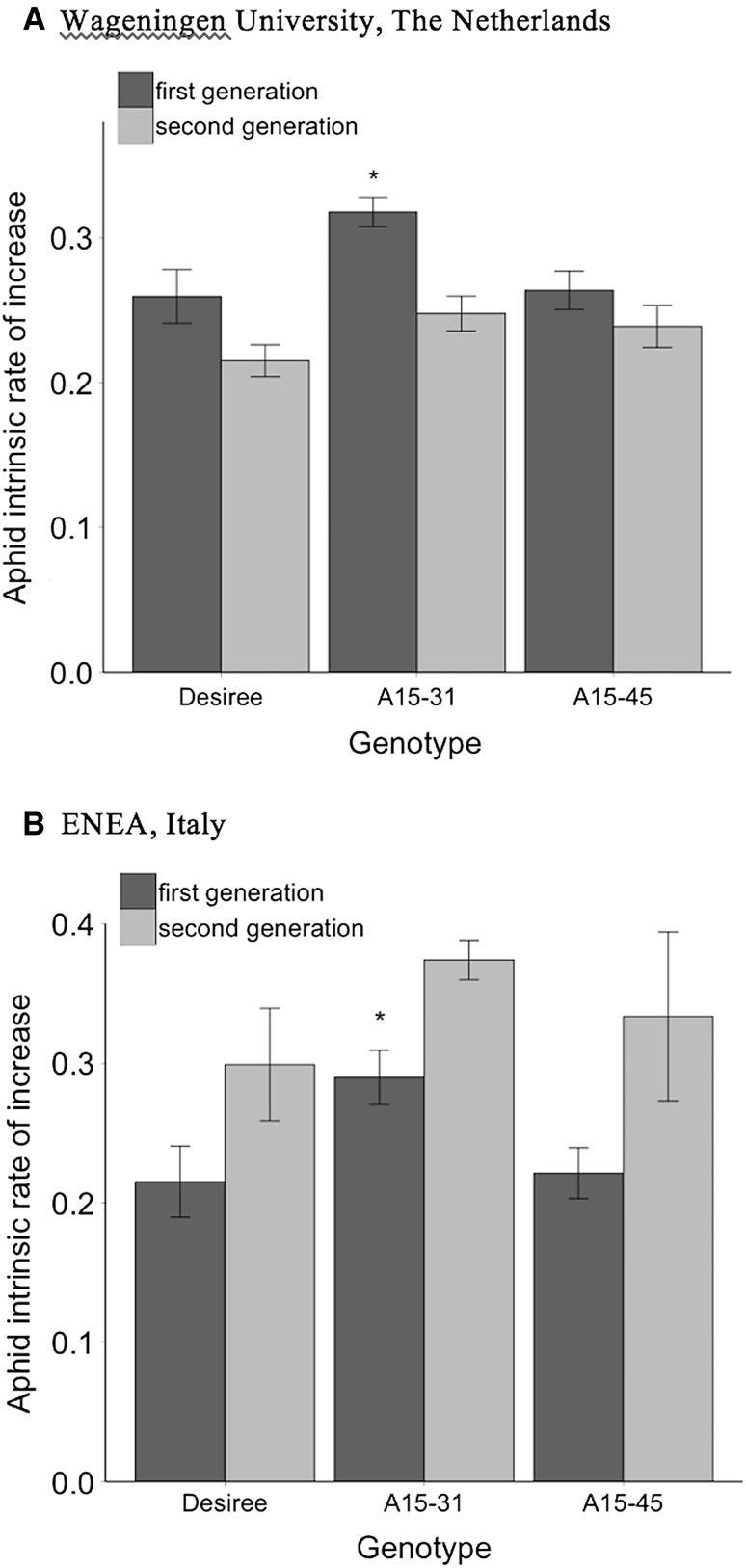
Mean aphid intrinsic rate of increase (±SE) on *Solanum tuberosum* isogenic cultivar Désirée, compared to genetically modified events A15-31 and A15-45, for two aphid generations in **a** at WUR Laboratory of Entomology and **b** at ENEA laboratory. *Asterisk* (*) indicates significant differences from the isogenic cultivar in the first generation

In the first generation, aphid intrinsic rate of increase was generally higher on all GM events than on the non-transformed Désirée plants, though the only events significantly differing from Désirée were the transgenic event A13-17 (*p* = 0.0122) and the cisgenic event A15-31 (*p* = 0.0198; Fig. 1a). The trend of higher intrinsic rate of increase was no longer observed in the second generation, the events no longer differed from non-transformed Désirée (Figs. 1b, 2).

#### Reproducibility between laboratories

The higher rate of intrinsic increase in the aphid population in the first generation on the cisgenic event A15-31 was observed in the laboratories at WUR (Fig. 2a; *p* = 0.0138) and at ENEA (Fig. 2b; *p* = 0.0243). However, at WUR, aphids generally had a lower intrinsic rate of increase in the second generation (Fig. 2a; *p* = 0.0223); whereas in ENEA, it was generally higher in the second generation (Fig. 2b; *p* = 0.0177).

#### Aphid survival

**Fig. 3:**
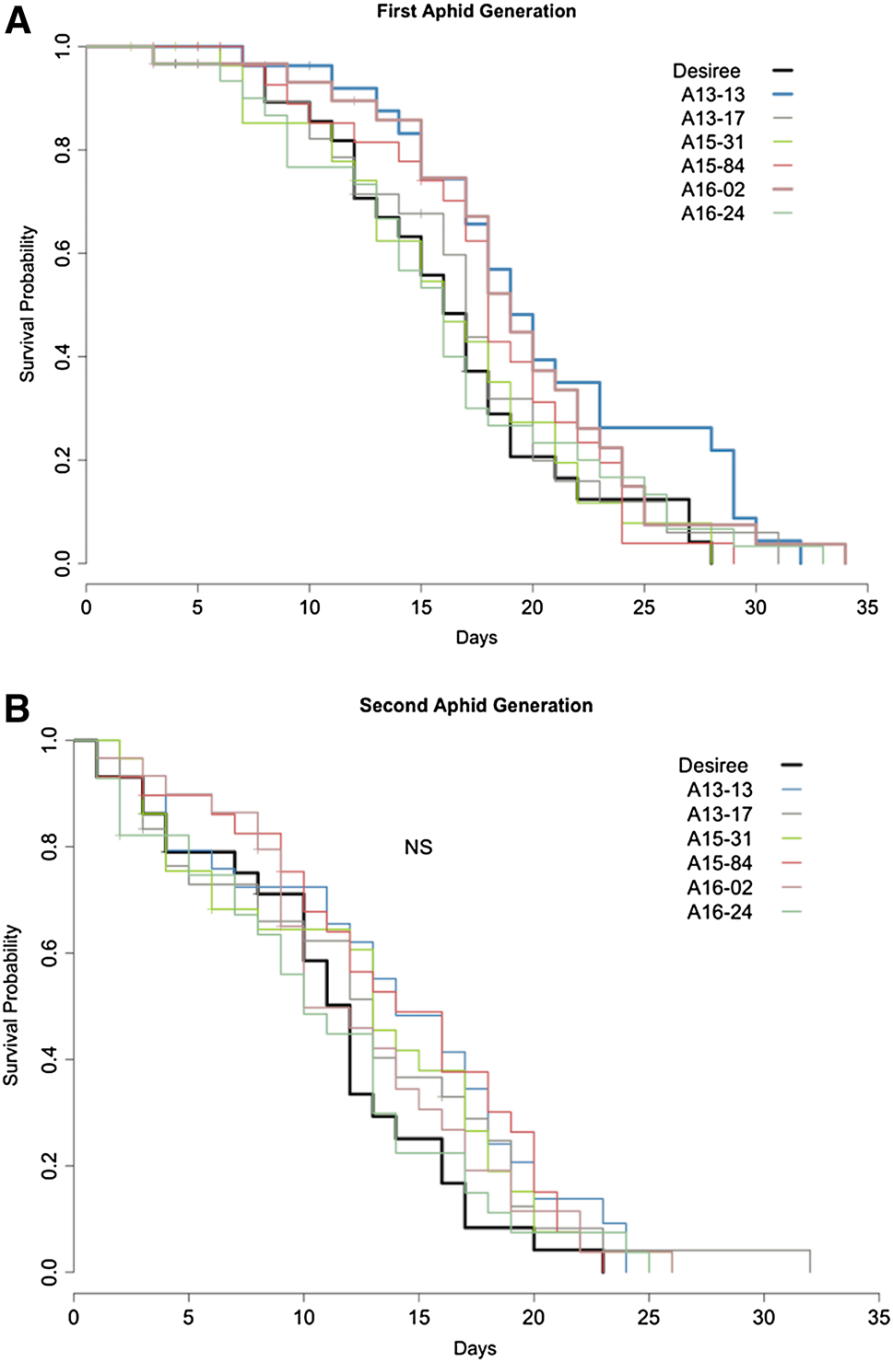
Probability of aphid survival per generation on *Solanum tuberosum* isogenic cultivar Désirée, compared to several genetically modified events. (Color figure online)

Probability of aphid survival over time also tended to be higher on the GM events as compared to the non-transformed Désirée, though only in the first generation significant differences were observed in one transgenic event A13-13 (*p* = 0.028) with a single *R* gene and one transgenic event with two *R* genes, A16-02 (*p* = 0.039) (Fig. 3a). In the second generation, there were no longer differences between the probabilities of survival of aphids on GM events compared to non-transformed Désirée (Fig. 3b).

No differences were found in the survival of aphids on Désirée compared to A15-31 or A15-45 at either WUR or ENEA (Appendix of Table 1).

### Baseline comparison with commercially available cultivars

**Fig. 4:**
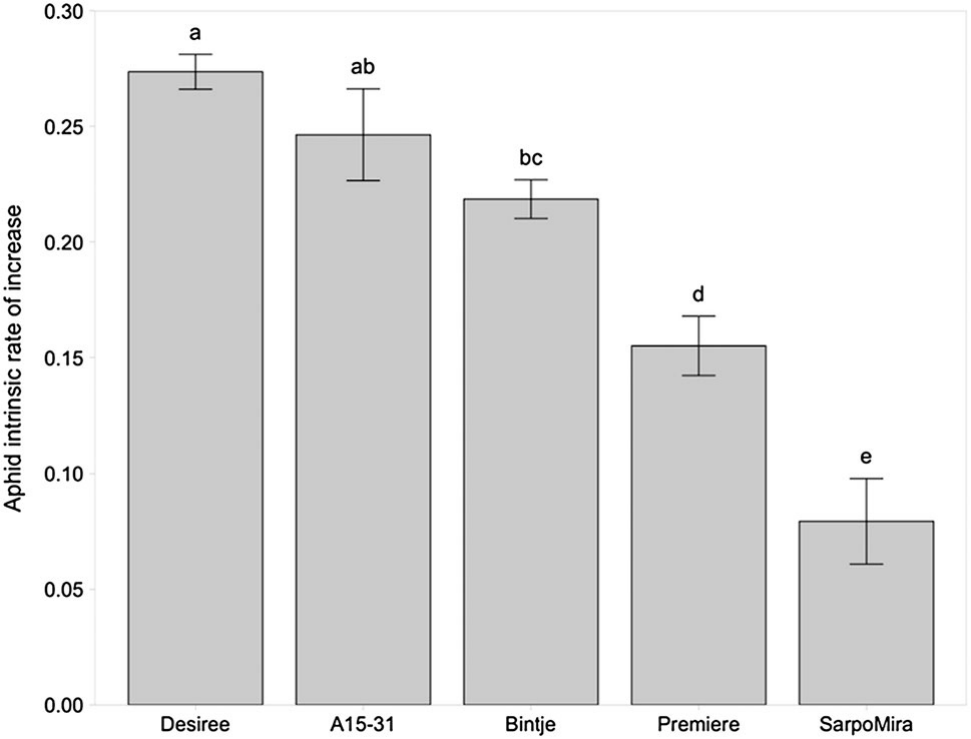
Mean aphid intrinsic rate of increase (±SE) on *Solanum tuberosum* isogenic cultivar Désirée, compared to a cisgenically modified event (A15-31), and three conventional cultivars Bintje, Première and Sarpo Mira. Different letters indicate significant differences between bars

In order to put these results into context of the differences found among conventionally bred and commercially available potato varieties, we tested aphids on three different varieties known to differ in level of resistance against *P. infestans*. Compared to Désirée, on the other three conventionally bred varieties, aphids had a lower intrinsic rate of increase (Désirée vs. Bintje: *p* = 0.002, and Désirée compared to Première and Sarpo Mira: *p* < 0.0001). When put into context of the conventionally bred varieties, there was no longer any difference between aphid rate of increase on the cisgenic event (A15-31) and Désirée (*p* = 0.1282). Although not different from the isogenic *P. infestans*-susceptible Désirée, the highly resistant cisgenic event (A15-31) also did not differ from the highly susceptible conventional variety Bintje (*p* = 0.1198) but aphids had significantly higher intrinsic rate of increase than on the highly *P. infestans*-resistant conventional variety Sarpo Mira (*p* < 0.0001; Fig. 4).

**Fig. 5:**
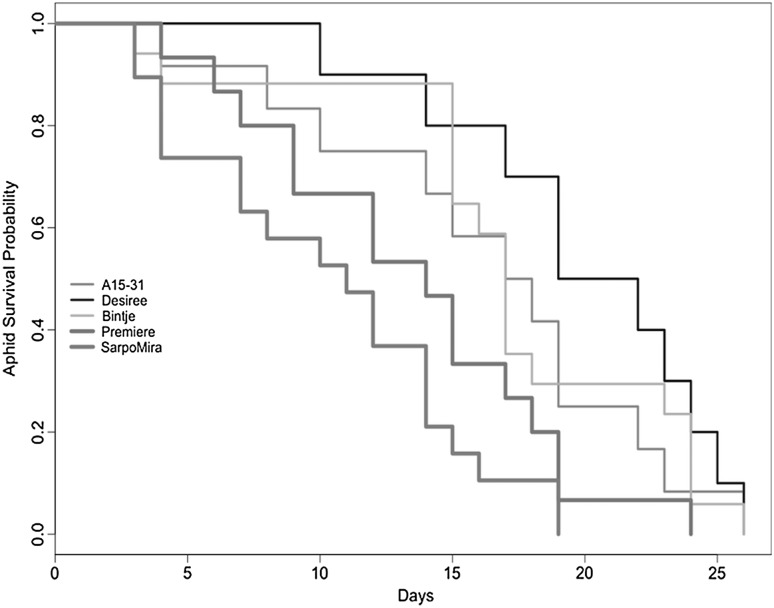
Probability of aphid survival per generation on *Solanum tuberosum* isogenic cultivar Désirée, compared to cisgenically modified event (A15-31), and three conventional cultivars Bintje, Première and Sarpo Mira. *Bold red* and *blue bold lines* indicate significant differences from the isogenic cultivar (Désirée). (Color figure online)

Probability of aphid survival did not differ between Désirée, Bintje and the cisgenic-resistant event A15-31 (Désirée vs. Bintje, *p* = 0.2919; Désirée vs. A15-31, *p* = 0.2225). However, aphid survival was significantly lower on Première (*p* = 0.0096) and Sarpo Mira (*p* < 0.0001; Fig. 5).

## Discussion

### Influence of selection markers, number of *R* genes, collateral effects and endpoint choice on detection of non-target effects

The results of our experiments show that genetic modification in potato for resistance to *P. infestans* through *R* gene insertion may have effects on non-target aphids in the first generation, yet these effects were no longer evident in the second generation of aphids. These effects cannot be attributed to marker gene use in the modification, since intrinsic rate of increase was higher both in a cisgenic and in a transgenic event. The differences found between events cannot be attributed to the number of *R* genes either, since survival probability was increased in events with both one and two *R* genes.

Interestingly, on the same event intrinsic rate of increase could be significantly higher, whereas survival did not differ. In our findings, significant effects on aphid life-history traits were never seen on both events transformed with the same construct. This brings to light the issue that detection of non-target effects depends on the measured endpoint (Charleston and Dicke 2008; Lövei et al. 2009). For example, in the case of the variety Bintje, it differed from Désirée in terms of aphid intrinsic rate of increase, yet had similar survival probability. Similarly, aphids on Désirée plants transformed to express enhanced chitinolytic activities showed increased population growth, while survival probability did not differ (Saguez et al. 2005). In the GM events, aphids had higher intrinsic rates of increase on A15-31 and A13-17, yet these were not the same events on which survival differed. Therefore, it is important to carefully select biologically relevant endpoints for testing in the greenhouse that can most closely translate to effect differences in the field. Considering several selected measurement endpoints when testing for environmental risk and non-target testing can be misleading if not all endpoints lead to differences in the same events. This considered, for the events tested at both WUR and ENEA, we came to comparable results with regard to both endpoints. Testing multiple endpoints in several events considerably strengthens the reliability of results of early tier risk assessments, but would require separate testable hypotheses and protection goals specific to each in order to reliably inform the assessment.

The location of the inserted *R* gene in the genome is the only difference between events transformed with the same construct. Since one event can influence aphid life-history traits, whereas another does not, we conclude that these are unintended effects associated with the location of insertion. These are known as position effects (Miki et al. 2009). These insertions may have occurred in a location that can affect interactions with insects such as defence response pathways. However, insertions usually result in loss of function rather than gain of function (Wang 2008). Loss of function effects are complemented by the three remaining copies in the tetraploid potato genome. A more likely explanation of the observed position effects could be a difference in expression level of the inserted *R* gene. Substantial differences in the expression level of the *Rpi*-*vnt1* gene are observed among different transformation events (J.H. Vossen, unpublished data). In this case, overexpression of a late blight *R* gene may have a trade-off with resistance to aphids. Generally, these results emphasize the usefulness of a pre-screening for position effects on relevant non-target insects before proceeding with an entire environmental risk assessment on a single modified event. These early tests can help detect possible position effects resulting from genetic modification.

### Detection of non-target effects over two insect generations

Our findings show that differences could be detected in the first generation of aphids feeding on GM events; however, these differences had disappeared in the second generation of aphids. Although transgenic resistance based on the expression of *Bacillus thuringiensis* (Bt) proteins has a very different mode of action, *Rhopalosiphum padi* aphids on Bt (transgenic) maize had higher performance in the first generation (Lumbierres et al. 2004). *Aphis gossypii* aphids also had higher intrinsic rates of increase on Bt cotton in the first, but not in the second or third generation (Liu et al. 2005). Since aphids were reared on the untransformed cultivar Désirée, it is possible that the effects seen in the first generation are a consequence of the aphids switching host plants rather than an effect of the transformation itself. This possibility can be tested in future experiments by rearing insects on an alternative host or on each of the test events separately.

The second generation of aphids was kept on the sample plants at WUR, yet at ENEA second-generation aphids were transferred to new plants. Although there were no differences in intrinsic rates of increase between genotypes detected in the second generation of aphids in either laboratory, the difference in performance of the second-generation aphids between experiments conducted at ENEA and Wageningen may have been caused by induced defence mechanisms since both generations were kept on the same plant in Wageningen. Feeding by conspecifics on the same plant can have negative effects on the life-history traits of *M. persicae*, due to systemic defence mechanisms of the plant (Dugravot et al. 2007).

Aphids are considered as good model organisms for understanding epigenetic effects (Srinivasan and Brisson 2012). The formation of winged offspring is a well-known epigenetic effect in aphids and can be triggered both pre- and post-natally by appropriate environmental cues (Brisson 2010; Sutherland 1969). The formation of sexual aphids is another example of epigenetic responses (Halkett et al. 2004). Although rapid epigenetic responses to changes in plant quality have not yet been studied, this could be an explanation for the changes we observed between rates of increase in two generations.

In aphids it is a natural situation for two generations (or more) to be present on the same plant. In our statistical models, we found in some cases that survival and rate of increase are significantly affected by the interaction of the factors ‘generation’ and ‘event’, which may also explain why observed effects are significant in the first, though not in the second generation. Additionally, the present paper allowed the set-up of a protocol that proved to be sensitive and reproducible and can be suggested as a standard for *in planta* studies with aphids in ERA.

### Significant effects in non-target tests should be compared to variation among conventionally bred varieties

Furthermore, our results point to the importance of comparing the differences found between GM events and the non-transformed variety to the variation among available conventional varieties in the agro-ecosystem. The concept of baseline variation has been documented before and is considered a necessary part of environmental risk assessment (EFSA 2010; Houshyani 2012). We show that when conventional cultivars are included in the comparison of the intrinsic rate of increase, the non-transformed and GM events no longer significantly differ, and rather the variation between conventionally bred varieties is much greater than between a non-transformed cultivar and derived GM events. Though significant effects may be found between the GM potato and its non-transformed progenitor when compared pairwise, this may be insignificant compared to the extent of variation already found between different conventionally bred potato varieties. In the case of our blight-resistant events, despite our sensitive assays, no biological relevance was detected for the non-target effect on aphids, since it proved to be in the range of effects present among available commercial varieties.

## Author contributions

JL, SA and JJAvL designed the research. JL, FB, PB, SM and EM conducted experiments. JHV contributed materials. JL and SA analysed data. JL, JHV, SA and JJAvL wrote, and all authors read and approved the manuscript.

## Appendix

See Tables 2, 3 and 4.
